# Postoperative adjuvant chemoradiation versus adjuvant radiation in surgically managed HPV-mediated OPSCC: a ten-year study using 8th edition AJCC pathologic staging

**DOI:** 10.3389/fonc.2026.1795834

**Published:** 2026-08-12

**Authors:** Avanti Verma, Ondrej Blaha, Soraya Fereydooni, Benjamin L. Judson, Ansley Roche, Sara I. Pai, Bruce H. Haughey

**Affiliations:** 1 Yale University School of Medicine, New Haven, CT, United States; 2 Division of Otolaryngology-Head and Neck Surgery, Department of Surgery, Yale University School of Medicine, New Haven, CT, United States; 3 Yale Center for Analytical Sciences, Yale University School of Medicine, New, Haven, CT, United States; 4 Department of Surgery, Faculty of Medical and Health Sciences, University of Auckland, Auckland, New Zealand; 5 Department of Otolaryngology-Head and Neck Surgery, University of South Florida, Tampa, FL, United States

**Keywords:** adjuvant chemoradiation, adjuvant radiation, human papillomavirus, metastatic lymph node, oropharyngeal cancer

## Abstract

**Introduction:**

HPV-mediated oropharyngeal squamous cell carcinoma (HPV+ OPSCC) is a disease associated with improved overall survival (OS). We explore the role and benefit of adjuvant chemoradiation in this population.

**Methods:**

9, 925 patients in the National Cancer Database (NCDB) who underwent definitive surgical treatment for HPV+ OPSCC followed by adjuvant treatment were included in the study. The primary outcome was OS.

**Results:**

4, 828 patients received adjuvant radiation (RT) and 5, 097 patients received adjuvant chemoradiation (CRT). There was no significant difference in OS between both groups. Increasing positive lymph node number increased the likelihood of patients receiving adjuvant CRT. In the proportional hazards model, patients with higher numbers of positive nodes had significantly reduced OS. The 5–6 positive node group was associated with a hazard ratio (HR) of 1.42 (CI: [1.15-1.75], p = 0.009) while > 6 positive nodes was associated with a HR of 2.19 (CI: [1.86-2.59], p < 0.001), when compared to 0–4 positive nodes. Multivariable analysis demonstrated no significant difference in OS between patients with ≤ 6 positive nodes with adjuvant CRT versus RT. The > 6 positive node group had significantly better OS with CRT versus RT.

**Conclusion:**

There was no observed difference in OS in surgically treated HPV+ OPSCC patients receiving adjuvant CRT versus adjuvant RT alone. Patients with higher positive node numbers were more likely to receive adjuvant chemotherapy, but no statistically significant survival benefit over RT was observed in patients with 0–6 involved nodes. Ongoing prospective trials should critically evaluate the role of adjuvant CRT in the postoperative management of HPV+ OPSCC.

## Introduction

The incidence of human papillomavirus-mediated oropharyngeal squamous cell carcinoma (HPV+ OPSCC) is rising, representing at least 70% of oropharyngeal cancers in the United States (1). HPV+ OPSCC has unique biological and clinical features; the 8^th^ Edition American Joint Committee on Cancer (AJCC) staging system included a separate staging system for HPV-mediated cases to refine prognosis (2). In contrast to HPV negative squamous carcinoma, HPV+ OPSCC is associated with improved survival, resulting in the investigation of de-escalation strategies to minimize treatment related toxicities.

While oropharyngeal cancers are treated with definitive chemoradiation with high cure rates, recent advances with minimally invasive surgery, such as transoral robotic and laser surgery, have provided an important de-escalation strategy. The Eastern Cooperative Oncology Group and the American College of Radiology Imaging Network (ECOG-ACRIN) clinical trial 3311, a multicenter randomized Phase II trial of transoral surgery with pathology-directed adjuvant treatment, confirmed the efficacy and safety of transoral surgery, followed by reduced dose intensity-modulated radiotherapy in intermediate risk patients (3). In this trial, the use of adjuvant chemotherapy in patients with high-risk pathologic features, such as positive margins and extranodal extension, was not investigated but remains a category 2B recommendation of the National Comprehensive Cancer Network (NCCN) (4) based on clinical trials that, however, make no distinction between HPV-mediated and non HPV-mediated diseases (5, 6).

To date, there has not been a large-scale study comparing outcomes with adjuvant radiotherapy versus adjuvant chemoradiotherapy. Small, single and multicenter cohort studies have found no difference in overall survival (OS) between patients treated with adjuvant radiation (RT) and adjuvant chemoradiation (CRT) (7, 8). The primary objective of this study was to analyze data from the largest cancer registry in the United States and perform a propensity score based, adjusted survival analysis to understand the impact of the addition of chemotherapy to adjuvant RT, versus RT alone, on OS for surgically treated HPV+ OPSCC. The secondary objective was to analyze the impact of the number of positive metastatic lymph nodes on OS in this cohort, relative to adjuvant treatment.

## Methods

### Selection criteria and variables

The data was derived from the National Cancer Database (NCDB) 2021 Participant User File (9). Since all data acquired and analyzed were de-identified, the study was exempt from a Yale University Institutional Review Board evaluation.

**Figure 1:**
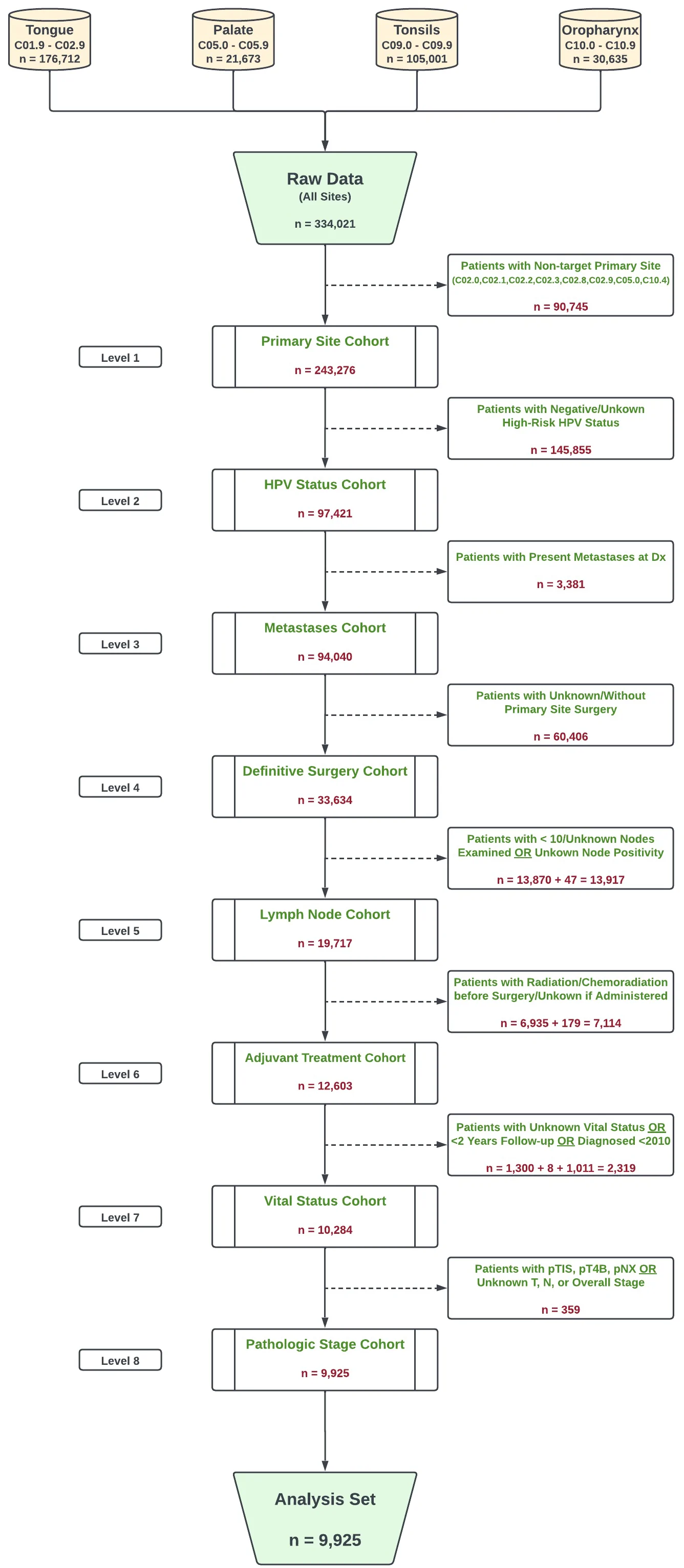
Flow diagram of the patient cohort, with application of inclusion and exclusion criteria.

All patients with HPV+ OPSCC were included who were treated with definitive primary site surgical resection (transoral or open) and neck dissection followed by no adjuvant therapy, adjuvant RT or adjuvant CRT between 2010 and 2020, with a minimum follow-up of 2 years and including all deaths before 2 years. Primary site determination was based on the *International Classification of Diseases for Oncology, 3*^rd^ *Edition*, which included (palatine) tonsil, base of tongue, overlapping lesion not otherwise specified (NOS) of the oropharynx, pharyngeal wall, and soft palate. Patients with high-risk HPV 16, HPV 18, or subtype NOS were considered HPV-mediated in patients diagnosed between 2010 and 2017. Patients diagnosed between 2018 and 2020 with a positive p16 immunohistochemistry were considered HPV-mediated, per updated NCDB criteria. Excluded patients had negative or unknown HPV status, unknown primary disease, pT4b or *in situ* primary site disease, < 10 lymph nodes examined following neck nodal surgery, systemic therapy and/or radiation received prior to surgery, distant metastatic disease at the time of presentation, and incomplete pathologic staging (**Figure 1**).

The target covariates of interest included age, sex, race, median income quartile, facility type, facility volume, travel distance, insurance status, Charlson-Deyo comorbidity score, histologic positive nodes, margin status of primary resection, AJCC 8^th^ edition pathologic T and N category, overall stage, lymphovascular invasion (LVI), and extranodal extension (ENE). ENE was stratified into microscopic (0.1-2.0 mm) and macroscopic (> 2.0 mm) ENE. The primary outcome was OS. Any missing or “unknown” ENE data were recorded and analyzed as a separate category.

All patients were staged according to the AJCC 8^th^ edition guidelines. 7^th^ edition staging of HPV+ OPSCC from the NCDB was converted to 8^th^ edition staging using a staging conversion tool (10). For T category, T4a and T4b were combined as T4. For N category, N1 and N2a were reclassified as N1. N2b, N2c, and N3 ≤ 4 lymph nodes were reclassified as N1 whereas > 4 lymph nodes was reclassified as N2.

### Statistical analysis

The data analyses were conducted using R, Version 4.3.2. The first step was to describe the study cohort. While demographic data were collected for patients who were observed following surgery (included in **Table 1**), the analysis was only performed on patients who received adjuvant treatment. A univariable analysis was performed, comparing patients treated with adjuvant CRT versus adjuvant RT. An unadjusted survival analysis between the two groups was conducted using the Kaplan-Meier (KM) estimator and the log-rank test. The Cox proportional hazard model was then fitted to assess the difference in OS between the two groups when controlling for relevant covariates.

**Table 1:** Demographic, pathologic, and staging data of the study cohort (no adjuvant and adjuvant treatment groups).

	Adjuvant therapy type			
Parameter	Total 14, 099 (100.0%)	No adjuvant 4, 174 (29.60%)	Radiation alone 4, 828 (34.24%)	Chemoradiation 5, 097 (36.15%)
Age at Diagnosis^†^ (median [range])	59.0 [23.0 90.0+]	61.0 [23.0 90.0+]	59.0 [28.0 90.0+]	58.0 [23.0 90.0+]
Age at diagnosis^†^ (n; %)				
≤ 50	2, 303	561 (24.36%)	810 (35.17%)	932 (40.47%)
51 70	10, 125	2, 920 (28.84%)	3, 469 (34.26%)	3, 736 (36.90%)
> 70	1, 671	693 (41.47%)	549 (32.85%)	429 (25.67%)
Sex (n; %)				
Male	12, 011	3, 399 (28.30%)	4, 119 (34.29%)	4, 493 (37.41%)
Female	2, 088	775 (37.12%)	709 (33.96%)	604 (28.93%)
Race (n; %)				
White	13, 284	3, 914 (29.46%)	4, 561 (34.33%)	4, 809 (36.20%)
Black	471	151 (32.06%)	154 (32.70%)	166 (35.24%)
Other	255	81 (31.76%)	86 (33.73%)	88 (34.51%)
Unknown	89	28 (31.46%)	27 (30.34%)	34 (38.20%)
Median income quartiles 2016-2020 (n; %)				
< $46, 277	1, 276	381 (29.86%)	421 (32.99%)	474 (37.15%)
$46, 277 $57, 856	2, 125	679 (31.95%)	666 (31.34%)	780 (36.71%)
$57, 857 $74, 062	2, 818	843 (29.91%)	946 (33.57%)	1, 029 (36.52%)
≥ $74, 063	5, 693	1, 629 (28.61%)	2, 105 (36.98%)	1, 959 (34.41%)
Missing	2, 187	642 (29.36%)	690 (31.55%)	855 (39.09%)
Facility type (n; %)				
Academic	9, 197	3, 007 (32.70%)	3, 265 (35.50%)	2, 925 (31.80%)
Non-Academic	4, 699	1, 112 (23.66%)	1, 500 (31.92%)	2, 087 (44.41%)
Missing	203	55 (27.09%)	63 (31.03%)	85 (41.87%)
Facility volume (n; %)				
> 90^th^ percentile	9, 797	3, 223 (32.90%)	3, 405 (34.76%)	3, 169 (32.35%)
≤ 90^th^ percentile	4, 302	951 (22.11%)	1, 423 (33.08%)	1, 928 (44.82%)
Treatment at >1 facility (n; %)				
Yes	3, 826	826 (21.59%)	1, 439 (37.61%)	1, 561 (40.80%)
No	10, 273	3, 348 (32.59%)	3, 389 (32.99%)	3, 536 (34.42%)
Travel distance (miles) (n; %)				
< 20	6, 126	1, 563 (25.51%)	2, 166 (35.36%)	2, 397 (39.13%)
≥ 20	5, 941	2, 014 (33.90%)	2, 020 (34.00%)	1, 907 (32.10%)
Missing	2, 032	597 (29.38%)	642 (31.59%)	793 (39.03%)
Insurance status (n; %)				
Private	8, 665	2, 276 (26.27%)	3, 124 (36.05%)	3, 265 (37.68%)
Medicare	3, 967	1, 444 (36.40%)	1, 249 (31.48%)	1, 274 (32.11%)
Medicaid	759	221 (29.12%)	223 (29.38%)	315 (41.50%)
Other Government	356	130 (36.52%)	113 (31.74%)	113 (31.74%)
Not Insured	223	63 (28.25%)	73 (32.74%)	87 (39.01%)
Unknown	129	40 (31.01%)	46 (35.66%)	43 (33.33%)
Total Charlson-Deyo score (n; %)				
0	11, 350	3, 256 (28.69%)	3, 963 (34.92%)	4, 131 (36.40%)
1	1, 973	628 (31.83%)	621 (31.47%)	724 (36.70%)
2	458	165 (36.03%)	145 (31.66%)	148 (32.31%)
3+	318	125 (39.31%)	99 (31.13%)	94 (29.56%)
Primary site (n; %)				
Tonsil	8, 821	2, 563 (29.06%)	3, 005 (34.07%)	3, 253 (36.88%)
Base of Tongue	4, 329	1, 285 (29.68%)	1, 517 (35.04%)	1, 527 (35.27%)
Overlapping Lesion/NOS	818	264 (32.27%)	276 (33.74%)	278 (33.99%)
Pharyngeal Wall	71	32 (45.07%)	13 (18.31%)	26 (36.62%)
Soft Palate	60	30 (50.00%)	17 (28.33%)	13 (21.67%)
Histologically positive nodes (#) (n; %)				
0	1, 901	1, 409 (74.12%)	368 (19.36%)	124 (6.52%)
1	5, 342	1, 878 (35.16%)	2, 014 (37.70%)	1, 450 (27.14%)
2	2, 848	439 (15.41%)	1, 303 (45.75%)	1, 106 (38.83%)
3	1, 432	179 (12.50%)	574 (40.08%)	679 (47.42%)
4	811	83 (10.23%)	255 (31.44%)	473 (58.32%)
5	492	57 (11.59%)	118 (23.98%)	317 (64.43%)
6	320	31 (9.69%)	61 (19.06%)	228 (71.25%)
7	207	21 (10.14%)	46 (22.22%)	140 (67.63%)
8	153	18 (11.76%)	22 (14.38%)	113 (73.86%)
9	107	8 (7.48%)	14 (13.08%)	85 (79.44%)
10	83	7 (8.43%)	15 (18.07%)	61 (73.49%)
>10	403	44 (10.92%)	38 (9.43%)	321 (79.65%)
Histologically positive nodes (#) (n; %)				
0	1, 901	1, 409 (74.12%)	368 (19.36%)	124 (6.52%)
1 4	10, 433	2, 579 (24.72%)	4, 146 (39.74%)	3, 708 (35.54%)
5 6	812	88 (10.84%)	179 (22.04%)	545 (67.12%)
> 6	953	98 (10.28%)	135 (14.17%)	720 (75.55%)
Histologically positive nodes (#) (n; %)				
0 4	12.334	3, 988 (32.33%)	4, 514 (36.60%)	3, 832 (31.07%)
5 6	812	88 (10.84%)	179 (22.04%)	545 (67.12%)
> 6	953	98 (10.28%)	135 (14.17%)	720 (75.55%)
Surgical margins of the primary site (n; %)				
No Residual Tumor	11, 360	3, 760 (33.10%)	4, 177 (36.77%)	3, 423 (30.13%)
Residual Tumor NOS	915	150 (16.39%)	212 (23.17%)	553 (60.44%)
Microscopic Residual Tumor	1, 289	166 (12.88%)	307 (23.82%)	816 (63.30%)
Macroscopic Residual Tumor	66	10 (15.15%)	13 (19.70%)	43 (65.15%)
Not Evaluable	185	35 (18.92%)	36 (19.46%)	114 (61.62%)
Unknown	284	53 (18.66%)	83 (29.23%)	148 (52.11%)
Surgical margins of the primary site (n; %)				
Negative	11, 360	3, 760 (33.10%)	4, 177 (36.77%)	3, 423 (30.13%)
Positive	2, 270	326 (14.36%)	532 (23.44%)	1, 412 (62.20%)
Unknown/Not Evaluable	469	88 (18.76%)	119 (25.37%)	262 (55.86%)
Lymphovascular invasion (n; %)				
Not Present	8, 682	3, 019 (34.77%)	3, 039 (35.00%)	2, 624 (30.22%)
Present	3, 634	693 (19.07%)	1, 245 (34.26%)	1, 696 (46.67%)
Unknown	1, 783	462 (25.91%)	544 (30.51%)	777 (43.58%)
AJCC 8 pT stage (n; %)				
0	235	62 (26.38%)	61 (25.96%)	112 (47.66%)
1	6, 753	2, 088 (30.92%)	2, 384 (35.30%)	2, 281 (33.78%)
2	5, 887	1, 788 (30.37%)	1.972 (33.50%)	2, 127 (36.13%)
3	911	192 (21.08%)	327 (35.89%)	392 (43.03%)
4	313	44 (14.06%)	84 (26.84%)	185 (59.11%)
AJCC 8 pN stage (n; %)				
0	1, 916	1, 419 (74.06%)	373 (19.47%)	124 (6.47%)
1	10, 363	2, 553 (24.64%)	4, 107 (39.63%)	3, 703 (35.73%)
2	1, 820	202 (11.10%)	348 (19.12%)	1, 270 (69.78%)
AJCC 8 overall p-stage (n; %)				
I	11, 317	3, 761 (33.23%)	4, 110 (36.32%)	3, 446 (30.45%)
II	2, 520	388 (15.40%)	677 (26.87%)	1, 455 (57.74%)
III	262	25 (9.54%)	41 (15.65%)	196 (74.81%)
Extranodal extension (n; %)				
None	8, 953	3, 451 (38.55%)	3, 764 (42.04%)	1, 738 (19.41%)
Micro (0.1 2.0 mm)	1, 943	227 (11.68%)	411 (21.15%)	1, 305 (67.16%)
Macro (> 2.0 mm)	862	113 (13.11%)	160 (18.56%)	589 (68.33%)
Present (NOS)	1, 713	205 (11.97%)	292 (17.05%)	1, 216 (70.99%)
Unknown	628	178 (28.34%)	201 (32.01%)	249 (39.65%)
Extranodal extension (n; %)				
None	8, 953	3, 451 (38.55%)	3, 764 (42.04%)	1, 738 (19.41%)
Micro	1, 943	227 (11.68%)	411 (21.15%)	1, 305 (67.16%)
Macro	862	113 (13.11%)	160 (18.56%)	589 (68.33%)
Unknown/NOS	2, 341	383 (16.36%)	493 (21.06%)	1, 465 (62.58%)
Survival status (n; %)				
Dead	1, 865	575 (30.83%)	488 (26.17%)	802 (43.00%)
Alive	12, 234	3, 599 (29.42%)	4, 340 (35.47%)	4, 295 (35.11%)
Follow-up (median [range])	56.48 [0.16 156.81]	49.83 [0.16 154.15]	57.45 [2.40 156.81]	62.62 [2.04 155.24]
Landmark survival (S; 95% CI)				
5-year	0.883 (0.878 0.889)	0.862 (0.850 0.874)	0.913 (0.905 0.922)	0.872 (0.862 0.882)
10-year	0.765 (0.752 0.779)	0.743 (0.714 0.773)	0.816 (0.796 0.837)	0.740 (0.719 0.761)

^†^ Upper limit for age is 90+.

In the descriptive analysis, continuous variables were expressed as a median value along with each variable’s range, whereas categorical variables were expressed as counts and percentages. A univariable comparison was conducted using either an independent t-test or chi-squared test of association. Multiple Cox model was summarized using hazard ratios (HR) along with their 95% confidence intervals (CI) from the maximum-likelihood estimator with Efron approximation for handling the ties. P-values from the likelihood ratio test of overall effect are provided for each variable in the model. Finally, a comparison between the adjuvant CRT and adjuvant RT groups was conducted using an Overlap weighting, propensity score-based method. The weights were derived from a logistic model that included all variables associated with the difference in treatment assignment. The following clinical variables were included in the model: age, surgical margins of the primary site, LVI, presence/number of positive nodes, and ENE. Estimated propensity scores were used to calculate weights for each observation, and comparison between groups was conducted using the weighted KM estimator and the weighted log-rank test. All reported tests were considered two-tailed, and a p-value of < 0.05 was considered to be statistically significant.

## Results

We identified 14, 099 patients with HPV+ OPSCC in the NCDB, diagnosed from 2010 to 2020, whose primary treatment was surgery (**Figure 1**). Median follow-up was 56.5 months, whereas maximum follow-up was 156 months. 4, 174 (29.6%) patients received no adjuvant therapy (a), 4, 828 (34.2%) patients received adjuvant radiation alone (b), and 5, 097 (36.2%) patients received adjuvant chemoradiation (c). The final study cohort included 9, 925 patients, by aggregation of categories (b) and (c) above. Demographic data, pathologic data, and AJCC 8^th^ edition category/staging are listed in **Table 1**. Though demographic data were collected for patients who received no adjuvant therapy to provide a broad view of treatment categories and outcomes, the univariable, multiple, and causal inference analyses were performed only for patients who received adjuvant therapy.

### Demographics

The median age at diagnosis was 59 years, and most patients were white (94.4%) and male (86.8%). Univariable analysis (**Table 2a**) revealed that age greater than 70, Black race, treatment at a facility volume ≤ 90^th^ percentile, Medicare/Medicaid insurance, and higher Charlson-Deyo scores were each associated with significantly worse OS (95% CI, p < 0.05). The highest income quartile was associated with increased OS.

**Table 2:** (A) Univariable survival analysis of the study sample (adjuvant treatment groups) for all patient and disease characteristics with HRs, their 95% CIs, and associated p-values. (B) Univariable survival analysis of the positive nodes and therapy interaction of the study sample (adjuvant treatment groups) with HRs, their 95% CIs, and associated p-values.

(A)				
		PH Cox model		
Parameter	Total 9, 925 (100.0%)	Unadjusted HR	95% CI	p-value
Age at diagnosis^†^ (median [range])	59.0 [23.0 - 90.0+]	1.048	(1.042; 1.054)	< 0.0001
Age at Diagnosis^†^ (n; %)				< 0.0001
≤ 50	1, 742 (17.55%)	Ref.		
51 - 70	7, 205 (72.59%)	1.745	(1.461; 2.085)	< 0.0001
> 70	978 (9.85%)	3.881	(3.142; 4.793)	< 0.0001
Sex (n; %)				
Male	8, 612 (86.77%)	Ref.	(0.711; 0.996)	0.0452
Female	1, 313 (13.23%)	0.842		
Race (n; %)				0.0030
White	9, 370 (94.41%)	Ref.		
Black	320 (3.22%)	1.611	(1.245; 2.083)	0.0003
Other	174 (1.75%)	1.038	(0.681; 1.582)	0.8626
Unknown	61 (0.61%)	0.820	(0.368; 1.829)	0.6282
Median income quartiles 2016-2020 (n; %)				< 0.0001
< $46, 277	895 (9.02%)	Ref.		
$46, 277 - $57, 856	1, 446 (14.57%)	0.804	(0.656; 0.985)	0.0351
$57, 857 - $74, 062	1, 975 (19.90%)	0.692	(0.569; 0.842)	0.0002
≥ $74, 063	4, 064 (40.95%)	0.537	(0.448; 0.644)	< 0.0001
Missing	1, 545 (15.57%)			
Facility type (n; %)				
Academic	6, 190 (62.37%)	Ref.		
Non-Academic	3, 587 (36.14%)	1.138	(1.018; 1.274)	0.0235
Missing	148 (1.49%)			
Facility volume (n; %)				
> 90^th^ percentile	6, 574 (66.24%)	Ref.	(1.162; 1.454)	< 0.0001
≤ 90^th^ percentile	3, 351 (33.76%)	1.300		
Treatment at >1 facility (n; %)				
Yes	3, 000 (30.23%)	Ref.		
No	6, 925 (69.77%)	1.025	(0.911; 1.153)	0.6810
Travel distance (miles) (n; %)				
< 20	4, 563 (45.97%)	Ref.		
≥ 20	3, 927 (39.57%)	1.013	(0.899; 1.140)	0.8350
Missing	1, 435 (14.46%)			
Insurance status (n; %)				< 0.0001
Private	6, 389 (64.37%)	Ref.		
Medicare	2, 523 (25.42%)	2.685	(2.387; 3.020)	< 0.0001
Medicaid	538 (5.42%)	2.744	(2.238; 3.365)	< 0.0001
Other Government	226 (2.28%)	1.403	(0.940; 2.094)	0.0973
Not Insured	160 (1.61%)	1.358	(0.879; 2.098)	0.1684
Unknown	89 (0.90%)	1.229	(0.658; 2.296)	0.5176
Total Charlson-Deyo score (n; %)				< 0.0001
0	8, 094 (81.55%)	Ref.		
1	1, 345 (13.55%)	1.767	(1.537; 2.032)	< 0.0001
2	293 (2.95%)	2.238	(1.733; 2.889)	< 0.0001
3+	193 (1.94%)	4.289	(3.339; 5.509)	< 0.0001
Primary site (n; %)				0.0020
Tonsil	6, 258 (63.05%)	Ref.		
Base of Tongue	3, 044 (30.67%)	1.011	(0.896; 1.140)	0.8637
Overlapping Lesion/NOS	554 (5.58%)	1.367	(1.086; 1.720)	0.0078
Pharyngeal Wall	39 (0.39%)	1.731	(0.822; 3.643)	0.1485
Soft Palate	30 (0.30%)	2.712	(1.352; 5.442)	0.0050
Histologically positive nodes (#) (n; %)				< 0.0001
0	492 (4.96%)	1.665	(1.293; 2.145)	0.0001
1	3, 464 (34.90%)	Ref.		
2	2, 409 (24.27%)	1.247	(1.059; 1.468)	0.0080
3	1, 253 (12.62%)	1.327	(1.091; 1.615)	0.0047
4	728 (7.34%)	1.587	(1.269; 1.985)	0.0001
5	435 (4.38%)	2.177	(1.705; 2.778)	< 0.0001
6	289 (2.91%)	2.051	(1.530; 2.749)	< 0.0001
7	186 (1.87%)	3.088	(2.280; 4.183)	< 0.0001
8	135 (1.36%)	2.915	(2.037; 4.171)	< 0.0001
9	99 (1.00%)	3.343	(2.272; 4.920)	< 0.0001
10	76 (0.77%)	4.376	(2.912; 6.577)	< 0.0001
>10	359 (3.62%)	4.124	(3.343; 5.087)	< 0.0001
Histologically positive nodes (#) (n; %)				
0 - 4	8, 346 (84.09%)	Ref.		
5 - 6	724 (7.29%)	1.761	(1.468; 2.112)	< 0.0001
> 6	855 (8.61%)	3.003	(2.613; 3.452)	< 0.0001
Surgical margins of the primary site (n; %)				< 0.0001
No Residual Tumor	7, 600 (76.57%)	Ref.		
Residual Tumor - NOS	765 (7.71%)	1.681	(1.409; 2.007)	< 0.0001
Microscopic Residual Tumor	1, 123 (11.31%)	1.334	(1.138; 1.565)	0.0004
Macroscopic Residual Tumor	56 (0.56%)	2.191	(1.315; 3.650)	0.0026
Not Evaluable	150 (1.51%)	1.422	(0.963; 2.100)	0.0771
Unknown	231 (2.33%)	1.341	(0.961; 1.871)	0.0845
Surgical margins of the primary site (n; %)				< 0.0001
Negative	7, 600 (76.57%)	Ref.		
Positive	1, 944 (19.59%)	1.489	(1.314; 1.687)	< 0.0001
Unknown/Not Evaluable	381 (3.84%)	1.374	(1.062; 1.777)	0.0157
Lymphovascular invasion (n; %)				< 0.0001
Not Present	5, 663 (57.06%)	Ref.		
Present	2, 941 (29.63%)	1.627	(1.446; 1.832)	< 0.0001
Unknown	1, 321 (13.31%)	1.011	(0.853; 1.198)	0.9020
AJCC 8 pT stage (n; %)				< 0.0001
0	173 (1.74%)	2.116	(1.486; 3.014)	< 0.0001
1	4, 665 (47.00%)	Ref.		
2	4, 099 (41.30%)	1.444	(1.275; 1.635)	< 0.0001
3	719 (7.24%)	2.480	(2.071; 2.970)	< 0.0001
4	269 (2.71%)	3.876	(3.087; 4.865)	< 0.0001
AJCC 8 pN stage (n; %)				< 0.0001
0	497 (5.01%)	1.373	(1.081; 1.744)	0.0094
1	7, 810 (78.69%)	Ref.		
2	1, 618 (16.30%)	2.451	(2.171; 2.766)	< 0.0001
AJCC 8 overall p-stage (n; %)				< 0.0001
I	7, 556 (76.13%)	Ref.		
II	2, 132 (21.48%)	2.404	(2.140; 2.700)	< 0.0001
III	237 (2.39%)	4.276	(3.417; 5.352)	< 0.0001
Extranodal extension (n; %)				< 0.0001
None	5, 502 (55.44%)	Ref.		
Micro (0.1 - 2.0 mm)	1, 716 (17.29%)	1.658	(1.436; 1.914)	< 0.0001
Macro (> 2.0 mm)	749 (7.55%)	2.276	(1.880; 2.756)	< 0.0001
Present (Micro/Macro - Unknown)	1, 508 (15.19%)	1.961	(1.696; 2.267)	< 0.0001
Unknown	450 (4.53%)	1.309	(1.000; 1.713)	0.0498
Extranodal extension (n; %)				< 0.0001
None	5, 502 (55.44%)	Ref.		
Micro	1, 716 (17.29%)	1.658	(1.436; 1.914)	< 0.0001
Macro	749 (7.55%)	2.275	(1.879; 2.755)	< 0.0001
Unknown/NOS	1, 958 (19.73%)	1.803	(1.574; 2.067)	< 0.0001
Therapy (n; %)				
Radiation	4, 828 (48.64%)	Ref.		
Chemoradiation	5, 097 (51.36%)	1.456	(1.301; 1.630)	< 0.0001
B				
		PH Cox model		
Parameter	Total 9, 925 (100.0%)	Unadjusted HR	95% CI	p-value
Nodes*therapy interaction				< 0.0001
0 - 4	8, 346 (84.09%)	Ref.		
5 - 6	724 (7.29%)	1.419	(0.940; 2.140)	0.0958
> 6	855 (8.61%)	4.136	(3.032; 5.643)	< 0.0001
Radiation	4, 828 (48.64%)	Ref.		
Chemoradiation	5, 097 (51.36%)	1.240	(1.088; 1.414)	0.0013
5 - 6 * Chemoradiation	545 (5.49%)	1.220	(0.769; 1.934)	0.3981
> 6 * Chemoradiation	720 (7.25%)	0.628	(0.443; 0.892)	0.0093

^†^ Upper limit for age is 90+.

In the multivariable analysis (**Table 3**), Black race was no longer associated with an increased HR, whereas female gender was associated with a decreased HR of 0.77 (CI: [0.64-0.93], p = 0.008). Older age, treatment at a facility with volume ≤ 90^th^ percentile, Medicare/Medicaid insurance, and increasing Charlson-Deyo scores all maintained significantly increased HRs with 95% CIs and p values < 0.001.

**Table 3:** Multivariable survival analysis, with nodes*therapy interaction, of the study sample (adjuvant treatment groups) for all patient and disease characteristics with HRs, their 95% CIs, and associated p-values.

Parameter	Distribution (n; %) 9, 925 (100.0%)	Adjusted HR	95% CI	p-value
Age at diagnosis				< 0.0001
≤ 50	1, 742 (17.55%)	Ref.		
51 - 70	7, 205 (72.59%)	1.484	(1.208; 1.824)	0.0002
> 70	978 (9.85%)	2.252	(1.722; 2.944)	< 0.0001
Sex				
Male	8, 612 (86.77%)	Ref.		
Female	1, 313 (13.23%)	0.774	(0.640; 0.936)	0.0082
Race				0.4512
White	9, 370 (94.41%)	Ref.		
Black	320 (3.22%)	1.261	(0.951; 1.671)	0.1078
Other	174 (1.75%)	1.004	(0.611; 1.649)	0.9881
Unknown	61 (0.61%)	0.810	(0.302; 2.173)	0.6756
Median income quartiles 2016-2020				0.0009
< $46, 277	895 (9.02%)	Ref.		
$46, 277 - $57, 856	1, 446 (14.57%)	0.904	(0.735; 1.113)	0.3412
$57, 857 - $74, 062	1, 975 (19.90%)	0.840	(0.686; 1.029)	0.0917
≥ $74, 063	4, 064 (40.95%)	0.709	(0.587; 0.856)	0.0003
Missing	1, 545 (15.57%)			
Facility type				
Academic	6, 190 (62.37%)	Ref.		
Non-Academic	3, 587 (36.14%)	0.919	(0.783; 1.079)	0.3026
Missing	148 (1.49%)			
Facility volume				
> 90^th^ percentile	6, 574 (66.24%)	Ref.		
≤ 90^th^ percentile	3, 351 (33.76%)	1.399	(1.189; 1.645)	0.0001
Insurance status				< 0.0001
Private	6, 389 (64.37%)	Ref.		
Medicare	2, 523 (25.42%)	1.890	(1.629; 2.192)	< 0.0001
Medicaid	538 (5.42%)	2.040	(1.614; 2.579)	< 0.0001
Other Government	226 (2.28%)	1.090	(0.695; 1.710)	0.7066
Not Insured	160 (1.61%)	1.001	(0.596; 1.683)	0.9966
Unknown	89 (0.90%)	1.010	(0.477; 2.135)	0.9800
Total Charlson-Deyo ccore				< 0.0001
0	8, 094 (81.55%)	Ref.		
1	1, 345 (13.55%)	1.486	(1.272; 1.736)	< 0.0001
2	293 (2.95%)	1.708	(1.291; 2.261)	0.0002
3+	193 (1.94%)	2.727	(2.075; 3.583)	< 0.0001
Primary site				0.3918
Tonsil	6, 258 (63.05%)	Ref.		
Base of Tongue	3, 044 (30.67%)	0.985	(0.858; 1.131)	0.8323
Overlapping Lesion/NOS	554 (5.58%)	1.121	(0.866; 1.452)	0.3862
Pharyngeal Wall	39 (0.39%)	1.422	(0.632; 3.199)	0.3947
Soft Palate	30 (0.30%)	1.898	(0.938; 3.840)	0.0746
Surgical margins of the primary site				0.0358
Negative	7, 600 (76.57%)	Ref.		
Positive	1, 944 (19.59%)	1.206	(1.048; 1.390)	0.0092
Unknown/Not Evaluable	381 (3.84%)	1.096	(0.796; 1.508)	0.5757
Lymphovascular invasion				0.0004
Not Present	5, 663 (57.06%)	Ref.		
Present	2, 941 (29.63%)	1.269	(1.109; 1.451)	0.0005
Unknown	1, 321 (13.31%)	0.914	(0.753; 1.109)	0.3622
AJCC 8 pT stage				< 0.0001
0	173 (1.74%)	1.394	(0.869; 2.236)	0.1682
1	4, 665 (47.00%)	Ref.		
2	4, 099 (41.30%)	1.320	(1.148; 1.518)	0.0001
3	719 (7.24%)	2.131	(1.747; 2.598)	< 0.0001
4	269 (2.71%)	2.845	(2.216; 3.653)	< 0.0001
Extranodal extension				< 0.0001
None	5, 502 (55.44%)	Ref.		
Micro	1, 716 (17.29%)	1.320	(1.112; 1.568)	0.0015
Macro	749 (7.55%)	1.696	(1.359; 2.117)	< 0.0001
Unknown/NOS	1, 958 (19.73%)	1.429	(1.214; 1.682)	< 0.0001
Nodes*therapy interaction				0.0074
0 - 4	8, 346 (84.09%)	Ref.		< 0.0001
5 - 6	724 (7.29%)	1.275	(0.801; 2.030)	0.3053
> 6	855 (8.61%)	3.686	(2.628; 5.171)	< 0.0001
Radiation	4, 828 (48.64%)	Ref.		
Chemoradiation	5, 097 (51.36%)	1.043	(0.890; 1.222)	0.6021
5 - 6 * Chemoradiation	545 (5.49%)	1.133	(0.674; 1.902)	0.6381
> 6 * Chemoradiation	720 (7.25%)	0.541	(0.371; 0.791)	0.0015

### Overall survival

**Figure 2:**
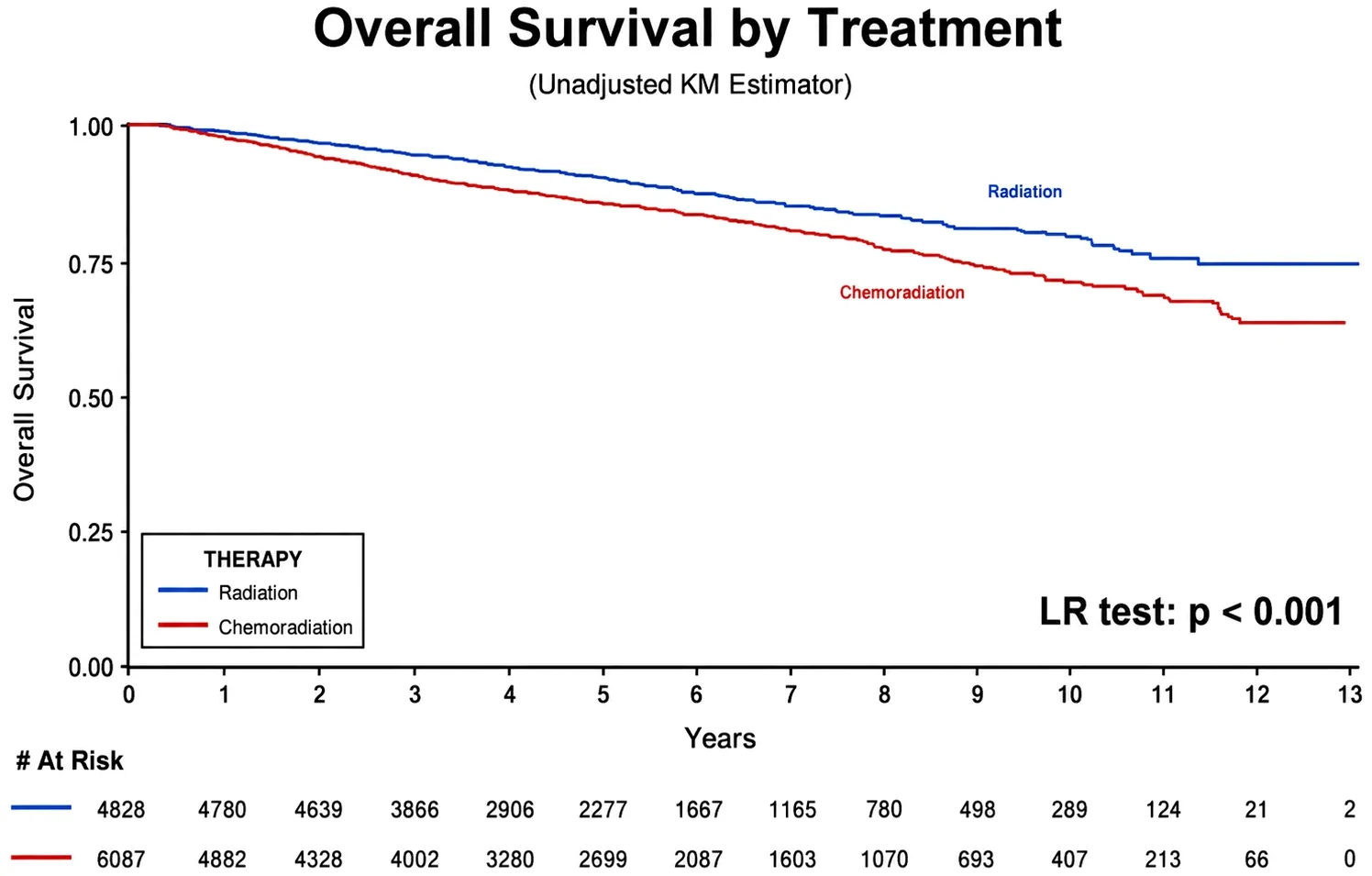
Overall survival in patients with HPV+ OPSCC who underwent primary surgery followed by adjuvant treatment, radiation versus chemoradiation.

**Figure 3:**
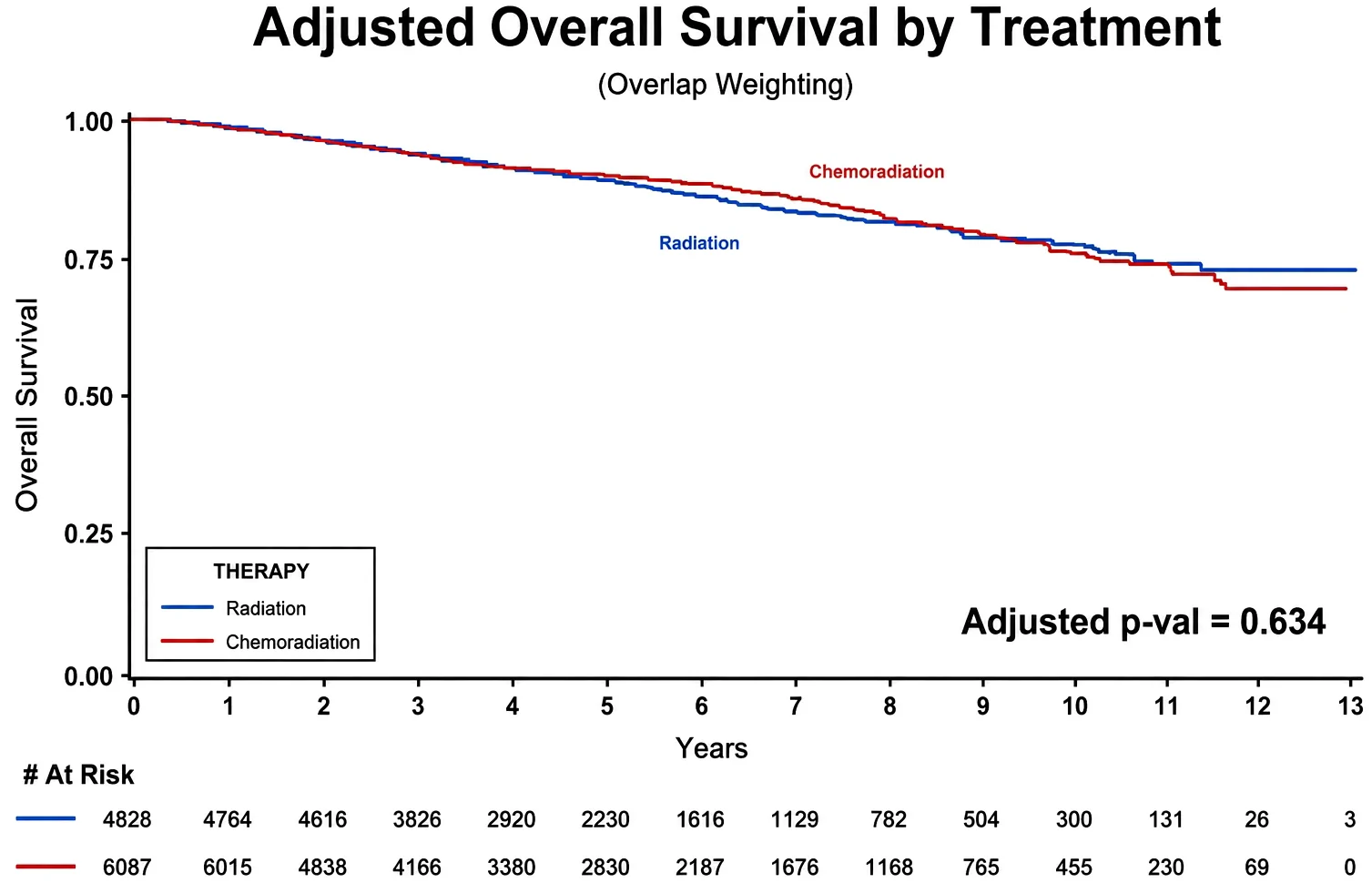
Adjusted survival in patients, using on overlap weighting approach, with HPV+ OPSCC who underwent primary surgery followed by adjuvant treatment, radiation versus chemoradiation.

The 5-year OS rate of the entire population was 88.3%, while the 10-year OS was 76.5% (**Table 1**). In the univariable analysis, adjuvant CRT was associated with a worse OS, HR of 1.46 (CI: [1.30-1.63], p < 0.001) (**Figure 2**), but multivariable analysis revealed no significant difference in OS between patients who received adjuvant RT and adjuvant CRT (HR = 0.96, CI: [0.83-1.11], p = 0.603) (**Figure 3**).

### Primary site

The most common primary tumor sites were tonsil (62.6%) and base of tongue (30.7%), with 47.9% of patients staged as pT1 and 41.8% of patients staged as pT2. Patients with higher T categories were more likely to be treated with adjuvant RT and adjuvant CRT. In the multivariable analysis, incremental escalation of pT categories from T1 to T4 at any site were significantly associated with worse OS. Anatomic primary site did not correlate with OS.

80.6% of patients underwent definitive surgical resection with negative margins, whereas 16.1% of patients had positive margins and 3.3% of patients had unknown or un-evaluable margins. Univariable analysis revealed that both macroscopic (> 2.0 mm) and microscopic (0.1-2.0 mm) positive margins were associated with worse OS (p = 0.003, p ≤ 0.001) compared to clear margins. In the multivariable analysis, positive margins remained significant in terms of worse OS (p = 0.006). In cases with LVI, 34.3% were treated with adjuvant RT and 46.7% were treated with adjuvant CRT. The presence of LVI was associated with a HR of 1.27 in the multivariable analysis (CI: [1.11-1.45], p ≤ 0.001).

### Nodal disease

Increasing tumor-involved lymph node number (1 through 10 and >10), were analyzed. Patients with increasing numbers of tumor-involved lymph nodes and a higher pN category were significantly more likely to receive adjuvant CRT than adjuvant RT. Univariable analysis of node number revealed an increase in HR as node number increased incrementally from 1 to 10 or more. However, similar HRs within node number groupings of 0-4 (reference value), 5-6 (HR 1.761), and > 6 involved nodes (HR 3.003) were observed with p-values <0.0001(**Table 2a, b**). AJCC pN2 category was associated with a HR of 2.45 (CI: [2.17-2.77], p<0.001) compared to pN1.

**Figure 4:**
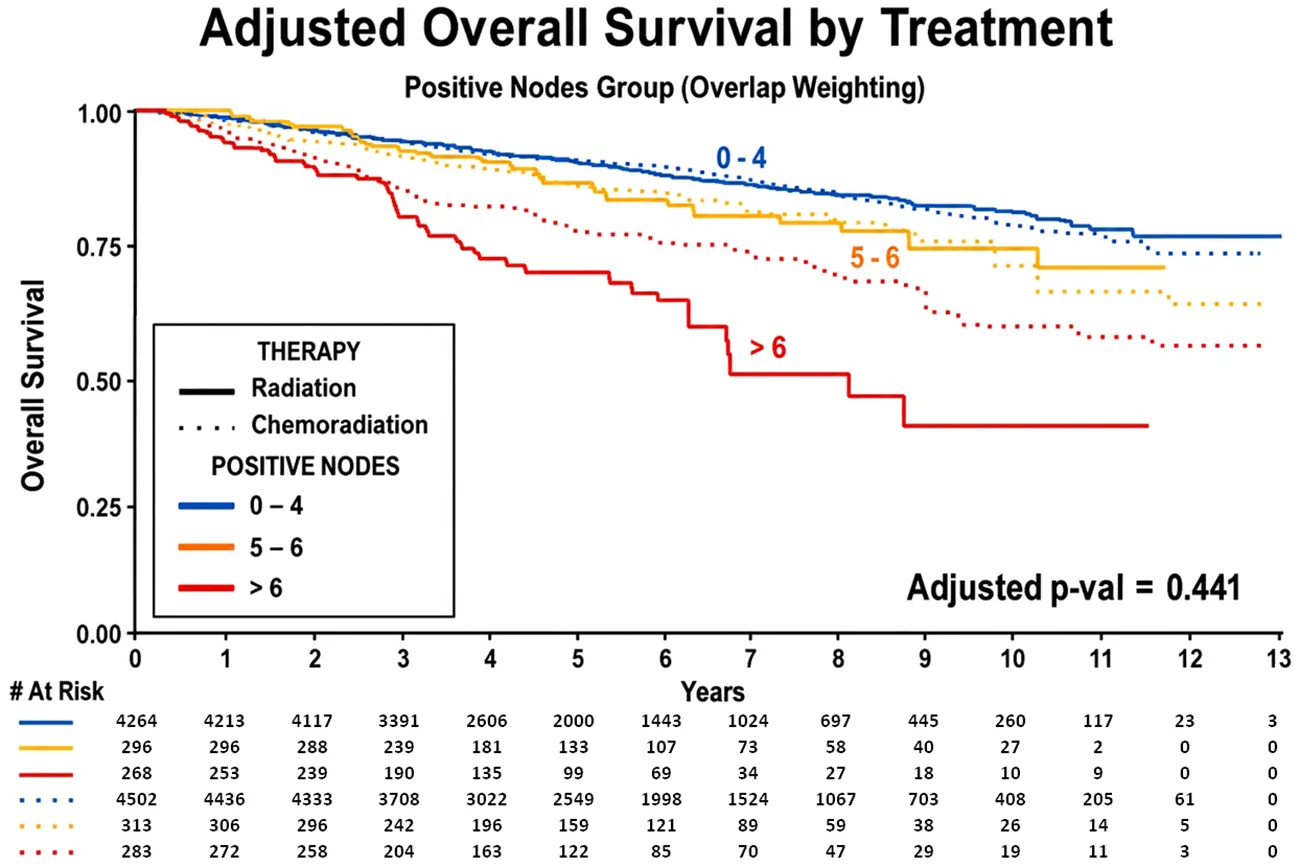
Adjusted survival in patients with HPV+ OPSCC who underwent primary surgery followed by adjuvant treatment, radiation versus chemoradiation, stratified by positive metastatic lymph node number in the following groups: 0-4 nodes, 5-6 nodes, > 6 nodes.

**Figure 5:**
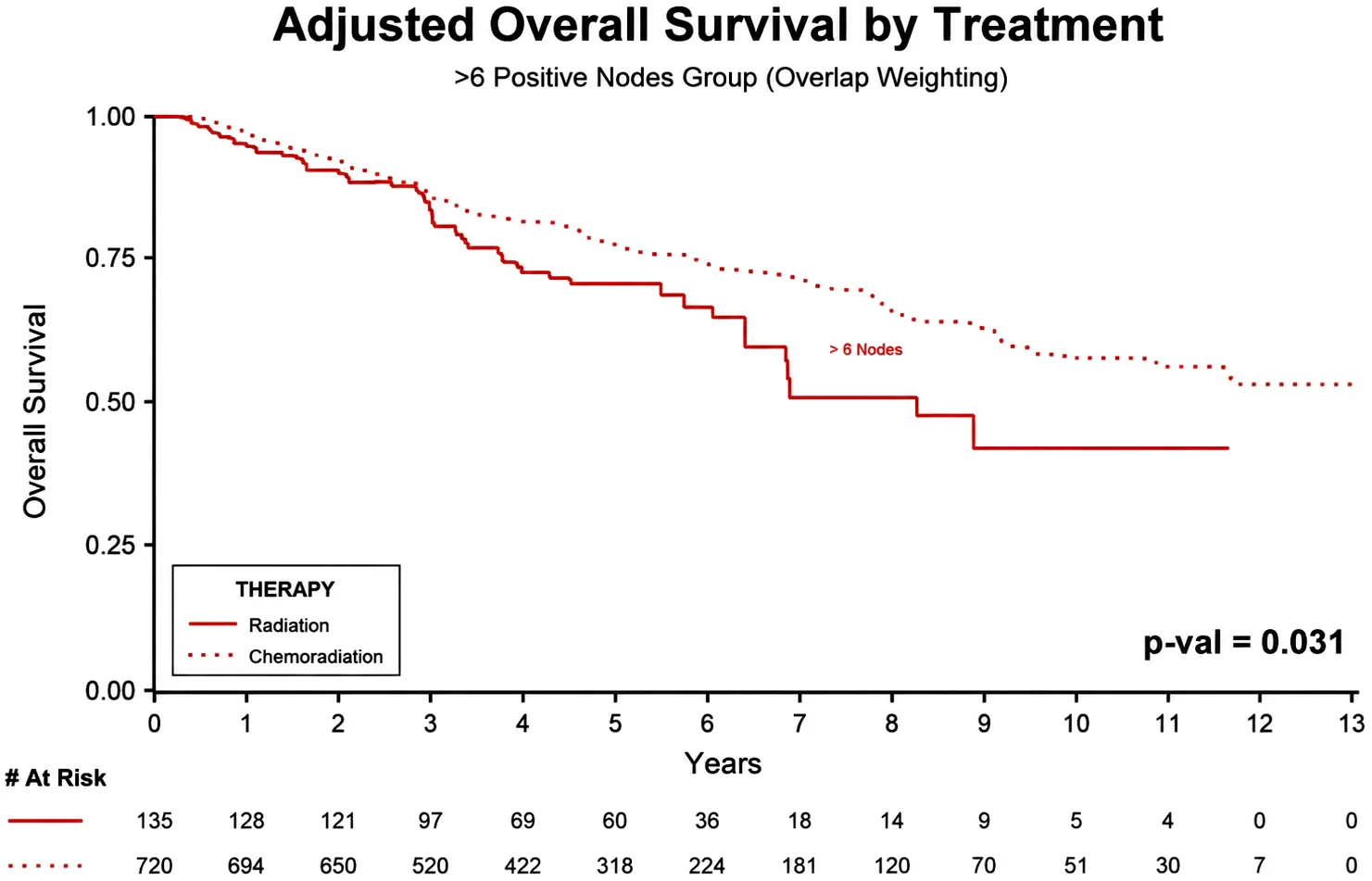
Adjusted survival in patients with HPV+ OPSCC who underwent primary surgery followed by adjuvant treatment, radiation versus chemoradiation, who had > 6 positive metastatic lymph nodes.

Multivariable analysis revealed that, compared to 0-4 positives nodes, 5-6 positive nodes were associated with a HR of 1.42 (CI: [1.15-1.75], p = 0.009), and > 6 positive nodes (n = 953, 6.8%) were associated with a HR of 2.19 (CI: [1.86-2.59], p <0.001). In the Overlap weighting analysis, with patients’ weights based on age and pathologic predictors (positive margins, LVI, positive node number, and ENE), there was no significant difference in OS between adjuvant RT and CRT among patients with 0–4 or 5–6 positive nodes (**Table 4**). However, in patients with > 6 positive nodes, improved OS was associated with the addition of adjuvant chemotherapy (**Figures 4**, **5**).

**Table 4:** Adjusted survival analysis of the positive nodes and therapy interaction using the overlap weights with HRs, their 95% CIs, and associated p-values.

		PH Cox model		
Parameter	Total 9, 925 (100.0%)	Unadjusted HR	95% CI	p-value
Nodes*Therapy Interaction				< 0.0001
0 - 4	8, 346 (84.09%)	Ref.		
5 - 6	724 (7.29%)	1.471	(0.940; 2.300)	0.0909
> 6	855 (8.61%)	4.165	(2.999; 5.785)	< 0.0001
Radiation	4, 828 (48.64%)	Ref.		
Chemoradiation	5, 097 (51.36%)	1.018	(0.876; 1.184)	0.8129
5 - 6 * Chemoradiation	545 (5.49%)	1.045	(0.626; 1.745)	0.8664
> 6 * Chemoradiation	720 (7.25%)	0.574	(0.391; 0.844)	0.0047

ENE was identified in 32% of patients. 67.2% of patients with microscopic ENE and 68.3% of patients with macroscopic ENE were treated with adjuvant CRT. ENE was associated with worse OS with significant differences in HRs between microscopic and macroscopic ENE in the univariable analysis. The multivariable analysis revealed a HR of 1.48 associated with the (aggregated) presence of ENE (CI: [1.28-1.71], p <0.001), where the reference level is absence of ENE (**Table 3**).

**Figure 6:**
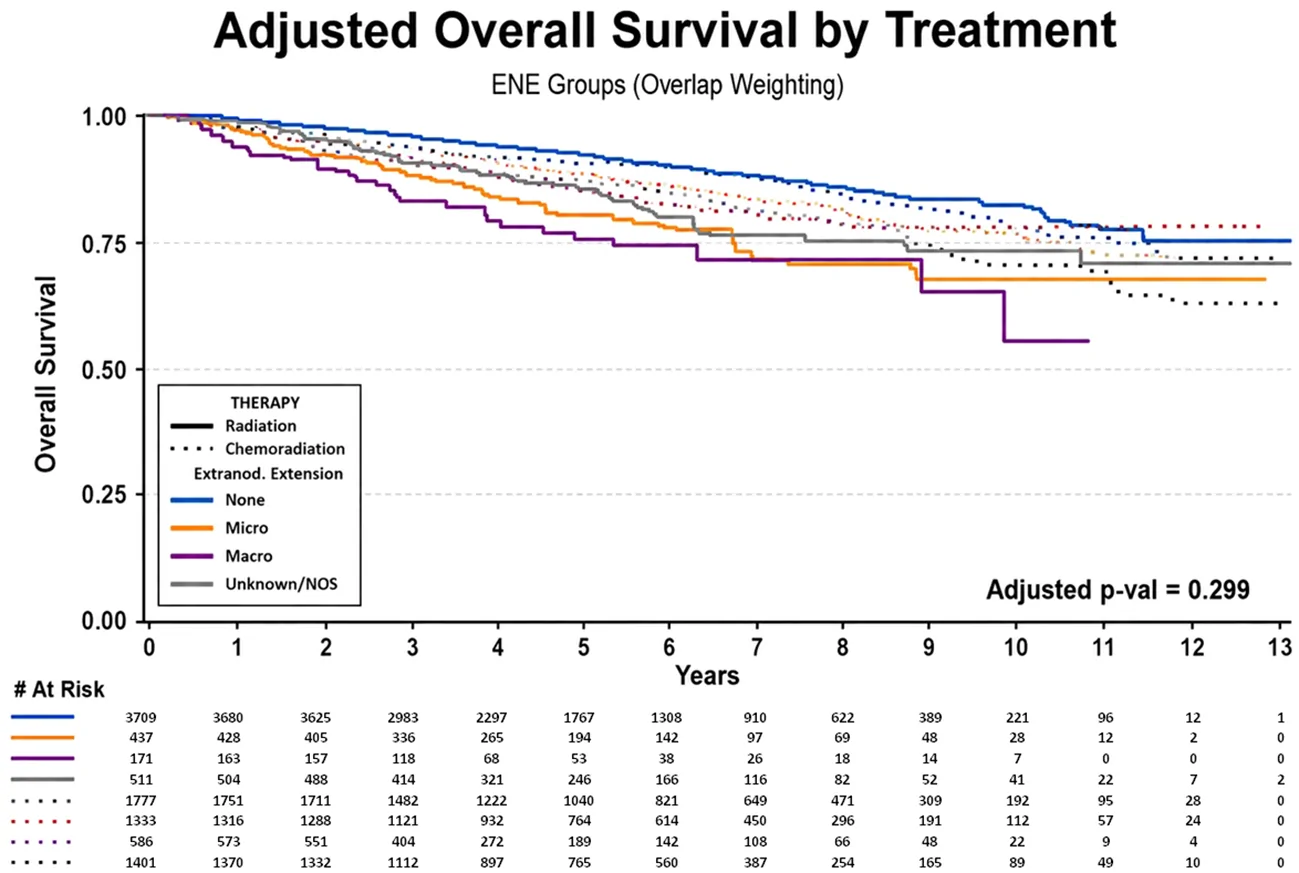
Adjusted survival in patients with HPV+ OPSCC who underwent primary surgery followed by adjuvant treatment, radiation versus chemoradiation, stratifed by extranodal extension (ENE).

**Figure 6** illustrates the respective survival curves associated with ENE groups, following the Overlap weighting analysis, which demonstrated no significant difference between microscopic and macroscopic ENE (**Figure 6**).

## Discussion

In surgically managed in surgically managed HPV+ OPSCC patients who had > 6 positive lymph nodes, postoperative adjuvant chemoradiation resulted in an improved OS than in a cohort that received adjuvant radiation alone. No difference was observed in HPV+ OPSCC patients with ≤ 6 positive lymph nodes. This is the largest reported cohort of HPV+ OPSCC patients followed over a 10-year period, re-staged with the AJCC 8^th^ edition, and the only study that investigates the impact of postoperative adjuvant chemotherapy on OS adjusted for its interaction with metastatic lymph node number. Our findings are supported by smaller studies that did not use AJCC 8^th^ edition staging. Sinha et al. studied a single institution cohort of 152 ENE positive patients (11) and Skillington et al. studied a single institution cohort of 195 surgical patients (7). Both studies found comparable 5-year disease-free survival between patients who received adjuvant radiation and chemoradiation. An et al., in their HPV+ OPSCC NCDB study, also showed identical survival outcomes with or without adjuvant chemotherapy, overall and in 132 propensity score-matched patients with ENE (12). While the present study is focused on increased node positivity rather than ENE, the propensity score-weighted analysis revealed similar OS among ENE groups.

With increasing age and Charlson-Deyo scores, there was a lower likelihood of treatment with adjuvant CRT and lower OS. An analysis of the NCDB by Chen et al. analyzed patients with intermediate risk HPV+ OPSCC < 70 years and ≥ 70 years of age, which demonstrated no OS benefit for those who received adjuvant RT versus CRT (13). This may be related to the increased risk of toxicity and acute adverse effects (≥ grade 3) seen in the elderly and patients with multiple co-morbidities who are treated with adjuvant CRT (6). While the NCDB does not distinguish between different chemotherapeutic agents, additional consideration should be taken when determining the benefit of adjuvant chemotherapy in older cancer patients.

Although our quantitative analysis did not include patients who underwent observation only, we did collect demographic data from this group. There was approximately a one-third distribution between the observation, adjuvant radiation, and adjuvant chemoradiation groups, which in part, reflected the distribution of patients in ECOG-ACRIN 3311 (3). 11% of patients were assigned to arm A (observation), while 28% and 30% were respectively randomized to arm B (50 Gy) and arm C (60 Gy). 31% of patients were accrued in arm D (66 Gy and weekly cisplatin). In our study, 29.6% of patients were observed following surgery, and this group had decreased OS compared to the groups that received adjuvant treatment (**Table 1**). The higher distribution of observed patients compared to ECOG 3311 could be attributed to those who were either unable to complete or refused adjuvant treatment. A single cohort study from a high-volume center reported that 13% of patients did not complete recommended adjuvant treatment (14). Costantino et al. found that a proportion of older patients (≥ 70 years) were less likely to receive adjuvant treatment if it was clinically recommended, with 12.9% who did not receive radiation and 16.5% who did not receive chemotherapy (15).

HPV+ OPSCC is a unique disease for which de-escalation of treatment can safely be achieved while maintaining long term OS (3). However, decisions about adjuvant chemotherapy can still be based on pathologic risk factors invoked prior to the discovery of the prognostic impact of HPV. One example is the assumption that multiple positive nodes, usually imaging-based, mandates triple therapy if a primary surgical approach were to be implemented. Triple therapy would then argue against the adoption of surgery as a primary treatment. To improve the use of surgery as a de-escalation tool, it is therefore important to re-evaluate the potential benefit versus toxicity of adding chemotherapy to postoperative adjuvant regimens. This study showed an improved OS with adjuvant CRT only in patients with > 6 positive lymph nodes. However, a prospective and randomized clinical trial would be needed to confirm if adjuvant chemotherapy provides no survival benefit in patients ≤ 6 positive lymph nodes. There are ongoing prospective investigations, such as the Postoperative Adjuvant Treatment for Human Papillomavirus-positive Tumors (PATHOS) clinical trial, which prospectively analyzes outcomes between adjuvant RT and CRT in patients with high-risk pathology.

There are inherent limitations in the retrospective design of this study, and further limitations with the use of NCDB data in which certain variables (details of tobacco/alcohol exposure, perineural invasion, surgical approach, radiation dose, chemotherapy agent and dose, performance status, toxicities related to treatment, swallow function, and quality of life measures) are not captured. Recurrent disease and cause of death data are also not in the NCDB, making progression-free and disease-specific survival, commonly used outcomes in HPV-mediated OPSCC, incalculable. Of the variables that were able to be captured, missing or unknown data for those variables were recorded and analyzed separately. Missing data is a limitation in any large, real-life database study which is dependent on the quality of data that has been submitted. Also, other variables such as T category are sometimes used to determine adjuvant treatment policies, although not formalized in most guidelines. We did not analyze the impact of adjuvant treatment relative to T category, although T category was included and adjusted for in the multiple, proportional hazards model.

Despite these limitations, however, the NCDB provides one of the largest prospectively assembled, real-world cancer databases available and is, in fact, a hallmark of quality control amongst those cancer centers that participate.

## Conclusion

In this large, retrospective study of surgically treated patients with HPV+ OPSCC from 2010 to 2020, OS benefit was observed with the administration of adjuvant CRT compared to adjuvant RT only in those patients with > 6 tumor-involved lymph nodes. In those with ≤ 6 involved lymph nodes, there was no observed OS benefit with the addition of chemotherapy to adjuvant RT. Although prospective studies are necessary and ongoing to specifically address the question of the role of adjuvant chemotherapy, the results of this study highlight the nuances and complexity of decision-making for adjuvant treatment in this unique and steadily expanding group of head and neck cancer patients.

## Data Availability

The original contributions presented in the study are included in the article/**Supplementary Material**. Further inquiries can be directed to the corresponding author.

## Supplementary material

Supplementary Table 5 Standardized mean differences before and after propensity score weighting.
