# Supplementary material for: Postoperative adjuvant chemoradiation versus adjuvant radiation in surgically managed HPV-mediated OPSCC: a ten-year study using 8th edition AJCC pathologic staging
Source: Front Oncol. 2026 Aug 12;16:1795834. doi: 10.3389/fonc.2026.1795834 (PMC13506416; doi:10.3389/fonc.2026.1795834)
Supplement: Supplementary file 1 [file Table1.docx]

Supplemental Table 5. Standardized mean differences before and after propensity score weighting
